# Health Journals

**Published:** 1873-07

**Authors:** 


### HEALTH JOURNALS.

A general desire is being evinced among all classes of community for a more thorough understanding of matters of physiology and hygiene. This inclination of the people is being encouraged by the numerous health journals that have sprung into existence within the past five years, nearly all of which are being liberally supported and eagerly sought for. Nearly every form of disease has been discussed, and appropriate advice given with reference to its management, or its prevention, until the public are becoming quite familiar with the laws of disease and the practice of hygiene. If any important organ of the body has been neglected in these dissertations upon health, we might particularize the eye, which seems to go almost entirely neglected, save by one or two little manuals upon the care of the eye, written by Drs. Jeffries and Williams. This field seems to be left entirely to the BISTOURY, which has furnished from one to two articles upon diseases of the eye and their management, in every issue, for the past ten years. We are glad to note that these articles are extensively read and frequently copied into other journals, encouraging us to continue them as long as we can render them instructive and timely. The article in the present number gives much valuable information relative to the selection of glasses, a procedure which is but little understood either by those requiring the use of glasses, or who keep them for sale. We ask for it a careful persual.

